# NPH: are we giving up on patients with temporary improvement post shunt?

**DOI:** 10.1186/2045-8118-12-S1-P51

**Published:** 2015-09-18

**Authors:** Simon Thompson, Claudia Craven, Patricia Haylock-Vize, Edward Dyson, Aswin Chari, Samir Matloob, Neekhil Patel, Syed Shah, Andrew Stevens, Huan Wee Chan, Jinendra Ekanayake, Ahmed Toma, Lewis Thorne, Laurence Watkins

**Affiliations:** 1 The National Hospital for Neurology and Neurosurgery, UK

## Introduction

Normal pressure Hydrocephalus (NPH) is predominantly treated with a ventriculoperitoneal shunt (VPS) resulting in improvement in the Hakim triad (mobility, cognitive function, urinary continence). There are a population of patients who experience an improvement in symptoms post shunt insertion followed by a subsequent deterioration in their condition in the proceeding months / years. At our institution, a large volume (min 40ml) CSF withdrawal is made via the shunt reservoir in these patients, measuring pre/post mobility and cognitive function. Comparison is then made between pre/post results and if a clear improvement is seen, VP shunt surgical revision is offered.

## Methods

A single centre retrospective audit. Medical notes of temporarily improved NPH patients, admitted for a VPS Tap test and subsequent shunt revision at our institution over the past four years were reviewed. Walking test assessed over a 10m course at baseline, post initial shunt, pre Tap test, post tap test and post shunt revision were compared. Subjective feedback from patient / family also assessed.

## Results

29 patients underwent tap tests via VPS shunt reservoir. No cases of shunt infection or sub-dural collections experienced post tap. 19 patients showed clinical improvement post Tap, 2 unable to withdraw CSF and subsequently underwent proximal catheter revision, 8 did not see improvement post tap and did not proceed with further neurosurgical treatment. Of the 19 positive results, 18 subsequently underwent shunt revision and 1 was lost to follow-up. All patients saw an improvement in symptoms post shunt revision including patients who had a proximal catheter blockage. 16 patients underwent insertion of Miethke Pro-SA valve post revision and 2 underwent insertion of Miethke Pro-GAV valve.

## Conclusion

Taping of VP shunt in NPH patients with suspected blocked / under functioning shunt is a safe procedure and shows an accurate predictive value for improvement post surgery.

